# The Role of Gut Microbiome in Sleep Quality and Health: Dietary Strategies for Microbiota Support

**DOI:** 10.3390/nu16142259

**Published:** 2024-07-13

**Authors:** Monika Sejbuk, Adam Siebieszuk, Anna Maria Witkowska

**Affiliations:** 1 Department of Food Biotechnology, Medical University of Bialystok, Szpitalna 37, 15-295 Bialystok, Poland; anna.witkowska@umb.edu.pl; 2 Department of Physiology, Faculty of Medicine, Medical University of Bialystok, Mickiewicza 2C, 15-222 Białystok, Poland; adam.siebieszuk@gmail.com

**Keywords:** microbiome, gut, sleep quality, diet

## Abstract

Dietary components, including dietary fiber, unsaturated fatty acids, and polyphenols, along with meal timing and spacing, significantly affect the microbiota’s capacity to produce various metabolites essential for quality sleep and overall health. This review explores the role of gut microbiota in regulating sleep through various metabolites such as short-chain fatty acids, tryptophan, serotonin, melatonin, and gamma-aminobutyric acid. A balanced diet rich in plant-based foods enhances the production of these sleep-regulating metabolites, potentially benefiting overall health. This review aims to investigate how dietary habits affect gut microbiota composition, the metabolites it produces, and the subsequent impact on sleep quality and related health conditions.

## 1. Introduction

Gut microbiota refers to the millions of microorganisms residing in the human gastrointestinal tract [1]. Its diversity significantly impacts health from the prenatal period and is influenced by numerous factors, including ethnicity and gender. In contrast, the gut microbiome is a broader concept that includes not only these microorganisms but also the metabolites they produce, their genetic material, and other environmental conditions [2]. From birth through the entire lifespan, the microbiome experiences dynamic changes that significantly impact health.

In full-term newborns, the composition of the gut microbiota varies with the mode of delivery and the type of feeding [3]. Natural childbirth is associated with colonization that mirrors the characteristics of the mother’s vaginal tract, dominated by bacteria such as *Lactobacillus*, *Prevotella*, or *Sneathia* spp. [4]. In contrast, a cesarean section leads to a different type of colonization, more akin to the microorganisms found on the mother’s skin and in the oral cavity, such as *Enterobacter hormaechei*, *Enterobacter cancerogenus*, *Haemophilus parainfluenzae*, *Haemophilus aegyptius*, *Haemophilus influenzae*, *Haemophilus haemolyticus*, *Staphylococcus saprophyticus*, *Staphylococcus lugdunensis*, *Staphylococcus aureus*, *Streptococcus australis*, *Veillonella dispar*, and *Veillonella parvula*. Importantly, caesarean births not only lack exposure to the vaginal microbiota, but also to the fecal microbiota [5].

The feeding method also influences the composition of the gut microbiota in neonates and later infants. Newborns and infants who are breastfed have a different composition of gut microbiota, consisting mainly of *Lactobacillus*, *Staphylococcus*, and *Bifidobacterium*. In contrast, formula feeding is associated with gut microbiota consisting mainly *Roseburia*, *Clostridium*, and *Anaerostipes* [6]. It has also been observed that feeding with an artificial formula accelerates the maturation of the gut microbiota and increases the prevalence of microorganisms that may contribute to inflammatory processes [6].

Generally, the first two years of life are marked by the most dynamic and intensive changes in the intestinal microbiota. In addition to encountering a vast array of microorganisms in their environment, infants experience a pivotal developmental period marked by the expansion of their diet to include solid foods. The introduction of solids drives rapid changes in structural and functional microbial diversity, shaping a gut composition that increasingly resembles that of an adult [7]. By 5 years of age, the foundation of the gut microbiota is established, determining its basic structure throughout later life. Finally, the composition of the intestinal microbiome stabilizes during puberty [7,8].

The composition of the microbiota is influenced by past infections and the use of antibiotics, particularly within the first two years of life [9], as well as by nonsteroidal anti-inflammatory drugs and proton pump inhibitors [7]. In later stages of life, diet, lifestyle, chronic stress, environmental exposures, and xenobiotics play significant roles in shaping the microbiota [7,10,11].

## 2. Method

A systematic search of the literature was conducted in PubMed base to identify studies relevant to the current review. The following search string was applied: (“gut microbiome “OR” short-chain fatty acids “OR” sleep and gut microbiome “OR” diet and microbiome “OR” circadian rhythms “OR” microbiome and circadian rhythms “OR” chronotype “OR” tryptophan metabolism “OR” serotonin production “OR” gamma-aminobutyric acid “OR” sleep disorders “OR” sleep disorders and dysbiosis of the gut microbiome “OR” dietary elements “OR” dietary elements and microbiome “OR” dietary fiber “OR” polyphenols “OR” fats “OR” western diet “OR” fructose “OR” saccharose “OR” meat “OR” alcohol”). We tried to limit the search for papers in the 5-year range; however, if no data were available in this range, studies from earlier years were also included.

## 3. Review

### 3.1. How Do Gut Microbiota Affect Host Circadian Rhythms?

Biological clocks are intricate systems within living organisms that facilitate responses to the passage of time, anticipate environmental changes, and regulate a variety of physiological processes. This concept encompasses all mechanisms, structures, and pathways involved in the measurement or perception of time, spanning from the cellular to the systemic level [12]. The circadian clock is the most specialized type of biological clock. In humans, it serves as a predominant, multi-level, and robust timing system that operates on a nearly 24 h cycle. It is synchronized by external signals such as light and food intake, which influence its phase [13,14]. Within the microbiota, which is predominantly composed of unicellular organisms, there may exist more rudimentary forms of biological clocks. The functions of these simpler, hypothesized timing systems can be observed in the rhythmic activity of the microbiome. [12].

Disturbances in the quantitative and qualitative composition of the gut microbiota result in dysbiosis, which can disrupt the bodily functions, and potentially lead to disease and sleep disturbances [15,16]. The interdependence between the intestinal microbiota and the host suggest their mutual influence on the regulation of the host’s circadian system and microbiome’s activity [12]. Pathogenic factors resulting from the modern lifestyles (including, among others, the Western diet, exposure to artificial light during nighttime hours, late eating, and irregular sleep–wake cycles) disrupt the relationship between the host’s microbiome and the host, leading to changes in the gut microbiome and the host’s circadian clock. These changes, in turn, can create a favorable environment for the development of sleep disorders, chronic inflammation, and civilization diseases [17].

One of the most significant factors influencing the rhythmic activity of commensal microorganisms is the host’s circadian clock. Persistent disruption of the circadian rhythm, due to alterations in dietary habits, the use of electronic devices, jet lag, shift work, or stress, has a detrimental impact on the composition of the gut microbiome [18,19]. However, this influence is not one-sided, as it has been demonstrated that the rhythmicity of the gut microbiota can also affect the functioning of the circadian clock [12,13]. In mice, for instance, prebiotic fiber supplementation positively regulates the circadian clock through the rhythmic production of short-chain fatty acids (SCFAs) by the microbiome [13]. This, along with other emerging findings, highlights a clear connection between the rhythmicity of the gut microbiome and the host’s circadian rhythm. Nevertheless, the precise nature of this relationship remains unclear, and further research is required to determine the extent to which the rhythmicity of the gut microbiota affects the host’s circadian rhythm [12,13].

Both the timing and composition of meals are essential elements in maintaining the proper functioning of the biological clock [20]. Furthermore, differences between day and night are observed in terms of the composition, location, and functioning of the gut microbiome, which is dependent on the host’s feeding cycle [19].

**Figure 1:**
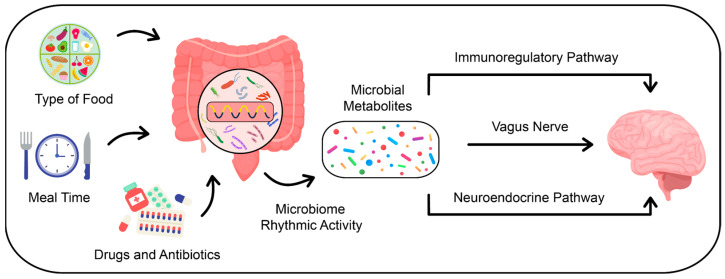
Visual representation of factors influencing the composition and activity of gut microbiota and its pathways of communication with the brain.

The brain and intestines are connected via the microbiome–gut–brain axis. The gut microbiota influences brain function through the following three pathways: immunoregulatory, neuroendocrine, and autonomic (Figure 1). In the immunoregulatory pathway, the microbiome interacts with immune cells, thereby influencing the levels of prostaglandin E2, cytokines, and the cytokine response factor. These processes modulate brain function [21]. In the context of the autonomic pathway, which is primarily constituted by the vagus nerve, sensory neurons of the intestinal muscular plexus form synaptic connections with motor neurons in the intestine. These motor neurons are involved in the neural regulation of intestinal hormone secretion and the control of intestinal motility patterns. The enteric nervous system also forms synaptic connections that link the vagus nerve with the brain, creating a pathway between the gut microbiome, vagus nerve, and brain [22]. Neurotoxic metabolites produced by the gut microbiome, such as ammonia and D-lactic acid, can negatively affect brain function, sleep quality, and stress responses through this pathway [23,24]. The neuroendocrine pathway provides another route by which the gut microbiome can influence the central nervous system and the hypothalamic–pituitary–adrenal axis, predominantly through the regulation of neurotransmitter secretion, including serotonin, cortisol, and melatonin [22].

Microorganisms colonizing the human gut are capable of producing various neurotransmitters and cytokines (e.g., short-chain fatty acids, dopamine, gamma-aminobutyric acid, 5-hydroxytryptophan, and melatonin). These metabolites can interact not only with the vagus nerve but also with the central nervous system by regulating enteroendocrine cells [25]. For instance, bacteria of the genera Lactobacillus and Bifidobacterium can secrete gamma-aminobutyric acid (GABA) [26], whose deficiency is positively correlated with sleep disorders. In patients with insomnia and depression, abnormal mRNA expression of gamma-aminobutyric acid is often observed [27].

As previously stated, the production of metabolites by the microbiota occurs rhythmically, exerting a significant influence on the host’s circadian rhythms and metabolism [28]. This provides further evidence of the impact of the microbiome on the host’s circadian and metabolic homeostasis. Furthermore, the gut microbiome is involved in the transformation of dietary choline into trimethylamine, which subsequently undergoes conversion in the liver. This process may potentially affect the expression of the host’s circadian clock genes in endothelial cells [29,30,31].

### 3.2. Circadian Rhythm, Chronotype, and the Interplay with Gut Microbiota: Insights into Sleep Regulation

Light is the primary factor responsible for the optimal functioning and precise fine-tuning of the human circadian clock, which is located in the suprachiasmatic nucleus of the hypothalamus [32,33]. However, numerous other stimuli and environmental signals can also substantially influence the circadian rhythm, including meal times, food type, exercise, body temperature, and even social interactions [33].

The gut–brain axis represents a complex biological system that allows for bidirectional communication between the brain and the gut. The gut microbiota plays a pivotal role in regulating this interaction, influencing various signaling pathways. The biological clock controls digestive physiology and gut barrier function, as well as modulating the expression of hormones and peptides. These processes regulate food intake through feelings of hunger and satiety [34]. The diversity and composition of the microbiota undergo daily changes that are strongly dependent on the time of day, the type of food consumed, and the use of fasting [35,36]. Therefore, abnormalities or simply improper eating habits can negatively affect the functioning of the biological clock [36]. The relationship between the gut microbiota and the circadian rhythm is bidirectional, with both gut microbiota disruption and circadian rhythm disturbances, affecting each other reciprocally [37].

The concept of chronotype refers to an individual’s circadian phenotype, encompassing their temporal preferences for wakefulness, activity, and sleep. Depending on the internal circadian rhythm, morning, and evening chronotypes can be distinguished. These can be further divided into extreme and moderate chronotypes. Additionally, a third type, neither-types, can be identified [38,39]. Approximately 40% of the adult population can be classified as morning and evening chronotypes, while approximately 60% are neither-types, which do not fit into the typically characterized categories. These individuals tend to maintain flexibility in their sleep and activity schedules, being more adaptable to different times of the day depending on the situation or preference [40].

Morning-type individuals tend to fall asleep and wake up early, closely aligning their sleep patterns with sunrise and sunset times. They typically experience their peak mental and physical performance in the early hours of the day. Conversely, evening-type individuals tend to go to bed and wake up much later, with their highest mental and physical performance occurring in the second half of the day. Nevertheless, it is more probable that they will encounter difficulties in level of alertness in the morning [40,41].

The gut microbiome may be involved in the discomfort associated with waking up in the morning. It has been observed that individuals with an evening type exhibit higher counts of Enterobacteriales and Enterobacteriaceae compared to morning types [42]. Bacterial strains that may be associated with longer sleep duration include *Lachnospiraceae*, *Odoribacter*, *Victivallaceae*, *Lentisphaerae*, and *Lentisphmulaeria* [42].

The microbiota–gut–brain axis has been demonstrated to impact circadian rhythms and, thus, the brain’s response to melatonin. For example, individuals with a higher abundance of *Selenomonadales* and *Negativicutes* may be at increased risk of insomnia [42]. Conversely, bacteria such as *Anaerofilum* and the order *Enterobacteriales* can affect the clock genes of the intestinal epithelium, thereby increasing the odds of the evening chronotype, which is associated with higher body weight [42]. Therefore, it is crucial to conduct further analyses that will extend the common understanding of the enormous impact of the gut microbiome on overall health.

### 3.3. Gut Microbiota-Derived Metabolites and Their Influence on Sleep Patterns

**Figure 2:**
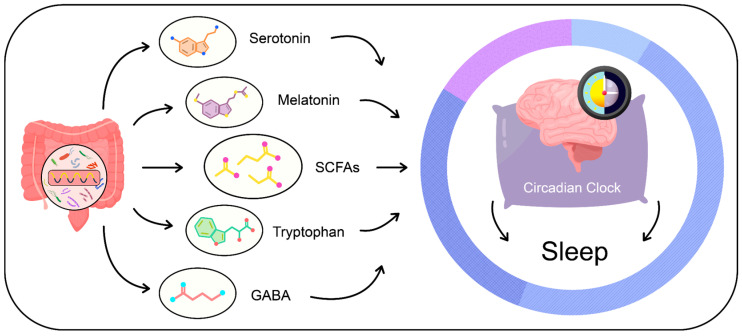
Metabolites produced by the gut microbiota.

The gut microbiota is recognized for its production of metabolites that can affect the quality of sleep. Key compounds among the metabolites directly and indirectly produced by the gut microbiota include short-chain fatty acids, serotonin, melatonin, and GABA [43,44,45,46] (Figure 2).

#### 3.3.1. Short-Chain Fatty Acids (SCFAs)

The small intestine, the longest segment of the digestive tract, is specialized in the processes of digestion, emulsification, and nutrient absorption, ensuring that only small amounts of simple carbohydrates, fats, and proteins reach the large intestine. In contrast, the opposite situation is observed with complex carbohydrates, such as dietary fiber, which are not digested in the small intestine but are instead metabolized in the large intestine by the microbiota [46]. Undigested dietary fiber serves as a vital nutrient for the gut microbiota, impacting its composition, abundance, and functionality [47]. Dietary fiber consists of carbohydrate polymers containing ten or more monomeric units that resist hydrolysis by endogenous enzymes in the human small intestine [48]. Another category of dietary fiber, commonly known as prebiotics, refers to substrates selectively utilized by host microorganisms, conferring health benefits [49]. While the majority of prebiotics can be categorized as dietary fiber, it is important to note that not all prebiotics fall under this classification [49].

Dietary fiber is abundant in plant-based foods, including whole-grain products, vegetables, fruits, nuts, seeds, and legumes [50]. In the large intestine, it undergoes fermentation by the microbiota, resulting in the production of short-chain fatty acids (SCFAs), which are small organic monocarboxylic acids with a chain length of up to six carbon atoms [51]. SCFAs are primarily composed of acetate, propionate, and butyrate, with an approximate molar ratio of 60:20:20. Following production, SCFAs are absorbed by enterocytes, transported to the portal circulation and subsequently utilized by hepatocytes [51,52].

Short-chain fatty acids (SCFAs) contribute to gut health by improving and enhancing intestinal barrier integrity, mucus production, and serotonin release [53,54]. Furthermore, SCFAs such as acetate, butyrate, and propionate, have been identified in human cerebrospinal fluid, indicating their involvement in maintaining central nervous system homeostasis and blood–brain barrier integrity [55].

Butyrate, one of the SCFAs produced by the microbiota, is also naturally present in dairy products such as milk, butter, and cheese [56]. Butyrate is readily absorbed into the portal circulation and transported to the liver [57]. This is evidenced by the concentration gradient between systemic and portal butyrate concentrations. Administration of tributyrin, an ester composed of three molecules of butyric acid and glycerol, to mice resulted in a nearly 50% increase in non-rapid eye movement (NREM) sleep for four hours, confirming that butyrate may act as a signaling molecule that induces sleep [57].

In another study, patients were divided into two groups: a research group comprising of individuals with acute and chronic insomnia (aged 26 to 55, with a group size of 20) and a control group of healthy individuals (matched for age and gender, with a group size of 38). It was observed that the gut microbiota differed significantly between the two groups. Individuals in the research group exhibited lower diversity and richness of gut microbiota, including microorganisms responsible for the production of SCFA. In patients with chronic insomnia, a decrease in the number of *Faecalibacterium*, *Prevotella*, and *Roseburia*—known contributors to butyrate production—was also observed. This decline may also influence sleep quality and the occurrence of insomnia [58].

Individuals with elevated levels of SCFA in their feces are more likely to experience symptoms of insomnia. Interestingly, elevated SCFA in feces is also observed in individuals with obesity-related diseases [54,59,60]. Enterochromaffin cells present in the intestines contain a specific receptor responsible for binding SCFA, which may affect serotonin production [61]. Serotonin, a key neurotransmitter, can influence the brain through interactions between enterochromaffin cells in the intestines and afferent nerve fibers via synapse-like connections [61,62]. As previously stated, higher SCFA levels in feces are observed in obese individuals and those with sleep problems, which may be caused by gut microbiota dysbiosis and impaired intestinal barrier permeability [54,62]. Consequently, it is proposed that maintaining a healthy gut microbiota has a considerable impact on sleep quality.

Six types of bacteria, including *Lachnospiraceae UCG004* and *Odoribacter*, which are responsible for the production of SCFAs, have been demonstrated to aid in prolonging sleep duration [51]. SCFAs have been shown to influence sleep by affecting the production of serotonin and GABA, which is the primary inhibitory neurotransmitter in the nervous system. GABA plays a significant role in promoting sleep, as it is involved in the inhibition of systems responsible for arousal [42,63].

Research indicates that changes in the composition of the gut microbiota are observed in individuals with sleep disorders. In patients with insomnia, a reduction in the abundance of bacteria responsible for the metabolism of dietary fiber to SCFAs can be observed, while SCFAs may have a beneficial effect on sleep duration [54,57,58].

#### 3.3.2. Tryptophan Metabolism and Serotonin Production

Tryptophan is an essential amino acid that is primarily found in protein-rich foods, including cheese, milk, nuts, seeds, fish, and lean meats [44,47]. The majority of tryptophan is absorbed in the small intestine, while the unabsorbed part serves as a substrate for microorganisms in the colon [64]. In contrast, serotonin (5-HT) is a monoamine neurotransmitter that plays a pivotal role in a multitude of physiological processes, including circadian rhythms, thermoregulation, emotion control, cognitive function, and pain perception. Serotonin is a precursor to melatonin, an essential hormone that regulates the biological clock, particularly with regard to sleep–wake cycles [43]. The majority of serotonin is produced by enterochromaffin cells of the gut, which utilize the rate-limiting enzyme tryptophan hydroxylase for its synthesis [45,65]. It is important to note that the production of 5-HT in the gut is influenced by various factors, including nutrients, gut microbiota, host-derived signaling hormones, and peptides. These factors directly and indirectly affect immune response, nutrient metabolism, and gut homeostasis [45,66]. The myenteric plexus is another site where much smaller, but still significant, amounts of serotonin are produced [45,67].

Tryptophan hydroxylase (TPH) is a specific enzyme involved in the synthesis of serotonin. In the body, two isoforms of TPH can be identified—TPH1, which is primarily found in enterochromaffin cells of the gut, and TPH2, which is predominantly located in the central nervous system and serotonergic neurons [68,69]. Both TPH and the rate-limiting enzyme are involved in the production of serotonin, which is then stored in vesicles located in the apical membrane of enterochromaffin cells [70]. 

It is noteworthy that the gut microbiota may play a significant role in serotonin secretion through the modulation of the production of various metabolites. As previously discussed, one of these metabolites can be SCFAs [71,72], which have the potential to increase TPH1 production, thereby contributing to increased serotonin synthesis [45,70]. This mechanism is supported by other studies, including one in which SCFAs administered to the lumen of the rat colon near the cecum increased serotonin production [73]. In addition to the fact that commensal gut microbes can increase the production of 5-HT, pathogenic microbes may cause the opposite situation, contributing to the development of diseases by decreasing the production of 5-HT. For example, enteropathogenic Escherichia coli infection can lead to decreased activity and reduced expression of the intestinal serotonin transporter [74]. 

#### 3.3.3. Melatonin

Melatonin plays a pivotal role in regulating the sleep–wake cycle for diurnal species, including humans. This naturally produced sleep-promoting hormone is synthesized in response to changes in environmental light, with its peak production occurring in the darkness.

Melatonin exerts its effects on sleep–wake mechanisms by interacting with melatonin receptors, mainly MT1 and MT2, which are located on the surface of neurons in the brain. These receptors are particularly abundant in the hippocampus, hypothalamus, and basal ganglia. Activation of melatonin receptors modulates the release of neurotransmitters such as GABA, serotonin, and glutamate, affecting brain structures involved in sleep regulation. Melatonin exerts its effects on the circadian rhythm, regulates the sleep–wake phase, and influences sleep quality. Its levels peak at night, facilitating the process of falling asleep, and decrease in the morning, promoting the transition to the wakefulness phase. Therefore, melatonin plays a key role in synchronizing the internal biological clock with the light–dark cycle, supporting sleep homeostasis [75,76,77].

It is noteworthy that the production of melatonin by intestinal cells is approximately 400 times greater than that of the pineal gland. Furthermore, its secretion seems to be correlated with the frequency of food intake [78]. 

Patients suffering from insomnia and other sleep disorders often exhibit disturbances in the composition and function of the gut microbiota [79]. As previously stated, serotonin is a precursor of melatonin that is produced in enterochromaffin cells of the gut. The activity of these cells can be modulated by the microbiome [43,80,81]. Consequently, the gut microbiota may play a significant role in supporting optimal melatonin production, which, in turn, may have a beneficial effect on sleep regulation and other functions related to the circadian rhythm. It has also been observed that melatonin impacts the activity of putative biological clocks in certain intestinal bacteria, thereby affecting their overall functioning [82]. However, the mechanisms of these interactions are complex and require further research.

#### 3.3.4. Gamma-Aminobutyric Acid (GABA)

GABA is a non-protein amino acid and the predominant inhibitory neurotransmitter in the brain. It plays a role in stress relief and sleep regulation [83]. GABA causes hyperpolarization of the postsynaptic neurons and the generation of an inhibitory postsynaptic potential. This increases the negative charge inside the cell, making the neurolemma harder to depolarize and thus reducing the likelihood of generating nerve impulses [84,85]. 

It is notable that some research indicates that GABA supplementation may be beneficial in terms of sleep quality. In a study, providing insomnia patients with 300 mg of GABA per day for four weeks was associated with increased sleep efficiency and decreased sleep latency [86]. In another study, the use of mixtures containing GABA and L-theanine resulted in reduced sleep latency and an increase in both rapid eye movement (REM) and non-rapid eye movement (NREM) sleep phases [87].

It is important to note that the vagus nerve is one of the potential pathways through which GABA can interact with the gut–brain axis. For example, studies have demonstrated that administering *Lactobacillus*, a GABA-producing bacterium, to mice resulted in a reduction of depressive markers and anxiety-like behaviors, whereas such effects were not observed in mice subjected to vagotomy [27,88].

Dietary sources of GABA include sprouts of common beans and peas, rice, oats, wheat, spinach, potatoes, and many vegetables, though in relatively small quantities. The presence of this acid in foods can be enhanced by incorporating *Lactococcus lactis*, *Lactobacillus brevis*, and other lactic acid bacteria into the food production process [44]. Additionally, GABA is also produced in the brain from another neurotransmitter, glutamate [83]. Studies have demonstrated that certain strains of gut bacteria are capable of producing GABA. One method of synthesizing this neurotransmitter involves the use of the glutamate decarboxylase (GAD) system. The conversion of glutamate to GABA via an enzyme dependent on pyridoxal-5′-phosphate, encoded by the gadA or gadB gene, is a key step in this pathway. A preliminary study of the Integrated Microbial Genomes/Human Microbiome Project database indicates that there are 26 genera of bacteria that contain orthologs of the gadB gene, including Bacteroides, which are abundant in the human gut microbiota [83,89].

Commensal bacteria involved in GABA production include *Lactobacillus*, *Bifidobacterium*, and *Bacteroides*. It has been demonstrated that bacteria belonging to the Lactobacillus and Bifidobacterium strains can increase GABA concentrations in the enteric nervous system [26]. However, other bacterial strains, such as *Ruminococcaceae* and *Escherichia coli K12*, may utilize GABA and cause the breakdown of this compound [90,91,92]. It can be observed that the gut microbiota can influence the metabolism of GABA, and that gut dysbiosis may contribute to the occurrence of sleep disorders.

### 3.4. Complications Resulting from Sleep Disorders and Dysbiosis of the Gut Microbiome

Proper sleep plays a role in numerous physiological processes, and, thus, abnormalities in its patterns can increase the risk of adverse health outcomes [93,94]. Therefore, sleep disorders constitute a distinct class of disorders caused by changes in the circadian timing system, with insomnia and excessive daytime sleepiness being among their primary symptoms [44,95].

Initial studies conducted fifty years ago indicated a correlation between abnormal sleep duration and an elevated risk of mortality. A landmark investigation involving over a million American adults demonstrated that both overly short and excessively long periods of sleep could elevate the risk of death [96]. Further research has corroborated these findings, indicating that sleeping for 6 h or less, as well as more than 9 h, increases mortality risk [97,98,99,100].

Sleep disorders are associated with higher levels of two inflammatory markers in the body, namely CRP and IL-6 [101]. SCFAs produced by the gut microbiota can have anti-inflammatory properties, and studies have observed that patients with sleep disorders have lower counts of certain bacteria involved in the production of these compounds [58].

Obesity is frequently accompanied by sleep disorders and insomnia. Meta-analyses of several cohort studies indicate a correlation between short sleep duration and an increased risk of obesity [102,103,104]. Furthermore, poor sleep quality is associated with worse fat loss despite adherence to a calorie-restricted diet [105]. The development of obesity is also positively correlated with the occurrence of gut microbiota dysbiosis [106]. It is noteworthy that obese individuals exhibit a decreased presence of bacteria belonging to the genera *Akkermansia*, *Faecalibacterium*, *Oscillibacter*, and *Alistipes* [106]. Similarly, a reduction in the abundance of *Faecalibacterium* is observed in patients with chronic insomnia [58].

Reducing sleep time to 4–5 h per night for just one week has been demonstrated to impair glucose tolerance and decreased insulin sensitivity in tissues [107]. Additionally, habitual short sleep duration (between 4.5 and 6 h) has been linked to significantly higher levels of glycated hemoglobin in individuals with type 2 diabetes [108]. Furthermore, gut microbiota dysbiosis may also play a role in the development of carbohydrate metabolism disorders, affecting physiological processes such as pancreatic beta-cell dysfunction, abnormal lipid and glucose metabolism, and chronic inflammation [109]. The gut microbiota plays a crucial role in tryptophan metabolism. Proper processing of this compound can positively affect insulin release, lower glucose levels, and enhance anti-inflammatory potential [110,111]. Conversely, a six-week study found that increasing time in bed by one hour above habitual duration was associated with improved insulin sensitivity in tissues in healthy adults who were chronically sleep restricted [112]. Therefore, it is important to recognize that both sleep disorders and gut microbiota dysbiosis, which impacts sleep, can influence the development of abnormalities in the body’s carbohydrate metabolism [45,73,110].

Cardiovascular diseases are the leading cause of mortality worldwide with hypertension, heart failure, and arterial atherosclerosis representing some of the most prevalent forms of this category of disease [113]. Hypertension is a disease that is associated with a number of factors including disorders in intestinal functioning, altered connectivity between the digestive and nervous systems, and changes in the gut microbiome. Patients with hypertension exhibit a significant reduction in the richness and diversity of gut microbes, as evidenced by studies [114,115], and the ratio of *Firmicutes* to *Bacteroidetes* is significantly higher as reported in [116]. The composition of gut bacteria exerts a significant influence on blood pressure regulation, primarily through the metabolites these microbes produce. Some metabolites appear to be of particular influence, playing a key role in this regulatory dynamic. A disruption in the production of SCFAs can result from gut microbiota dysbiosis, which, in turn, can stimulate enterochromaffin cells to produce 5-hydroxytryptamine. This neurotransmitter can influence the 5-HT3 receptor of the vagus nerve, inhibiting the afferent activity of the vagus nerve from the gut to the brain. Furthermore, the release of 5-hydroxytryptamine into the bloodstream can also cause vasoconstriction, which may affect the elevation of arterial blood pressure [117]. A growing body of research indicates a connection between gut microbiome dysbiosis and its role in regulating vascular permeability, the state of inflammation, and blood pressure [118,119].

Gut microbiome disorders may contribute to the development of angiotensin II-induced hypertension and vascular dysfunction through the infiltration and inflammation of immune cells within the vessels [120]. A study involving the transplantation of fecal matter from a hypertensive donor into the intestines of mice demonstrated that the recipients exhibited elevated blood pressure [121]. A further study, a randomized controlled trial, demonstrated that a diet rich in polyphenols can significantly improve intestinal permeability in the elderly, increase the number of gut bacteria capable of digesting cellulose and producing butyrate, and reduce blood pressure [122]. In addition to gut microbiome dysbiosis, sleep disorders, including reduced quality and insufficient duration, also contribute to the occurrence of hypertension [123,124]. Consequently, a comprehensive approach to patient care, which considers multiple interrelated factors, including sleep disorders and the gut microbiota, is of paramount importance.

Another significant public health concern is the prevalence of mental disorders, which may also be linked to disturbed sleep and gut microbiota dysbiosis [125,126]. Sleep deprivation in humans and animals has been shown to result in alterations in the expression of clock genes, which, in turn, can influence neurobiological responses to stress. This may increase susceptibility to stress and elevate the risk of subsequent disorders [127]. The gut microbiota plays a pivotal role in the production of various neurotransmitters, including dopamine, tryptophan, GABA, SCFAs, and melatonin. These metabolites exert a profound influence on the functioning of both the enteric and central nervous systems [128]. Furthermore, disrupted GABA expression is frequently observed in individuals with insomnia and depression [129].

Alzheimer’s disease is the most prevalent neurodegenerative disorder affecting the central nervous system. The characteristic pathological mechanisms of Alzheimer’s disease trigger an inflammatory response, which ultimately results in apoptosis or necrosis of neurons, causing irreversible brain damage [130]. Certain bacterial strains, including *Escherichia*, *Lactobacillus*, *Saccharomyces*, and *Bacillus*, are capable of synthesizing a range of amino acids, including gamma-aminobutyric acid, 5-hydroxytryptamine, dopamine, butyrate, histamine, and serotonin. These amino acids may play a crucial role in regulating brain activity [131,132]. The gut microbiota of elderly individuals typically exhibits reduced diversity and lower bacterial levels, which consequently results in lower butyrate production [133]. This may contribute to the development of inflammation and the progression of cognitive function loss [134,135]. There is mounting evidence to suggest that the composition of gut microbiome can influence the severity of β-amyloid pathology and cognitive impairment. The gut microbiome may play a significant role in the pathogenesis of Alzheimer’s disease, with disturbances in the gut–brain axis being particularly noteworthy. A growing body of evidence suggests a correlation between inflammation induced by gut microbiome dysbiosis and Alzheimer’s disease [130,136]. Furthermore, sleep disorders have been linked to an increased risk of developing Alzheimer’s disease [137,138,139]. As previously presented, numerous studies indicate a relationship between the occurrence of sleep disorders and gut microbiome dysbiosis [60,77,140].

### 3.5. Dietary Elements and Nutritional Strategies Affecting Gut Health

**Figure 3:**
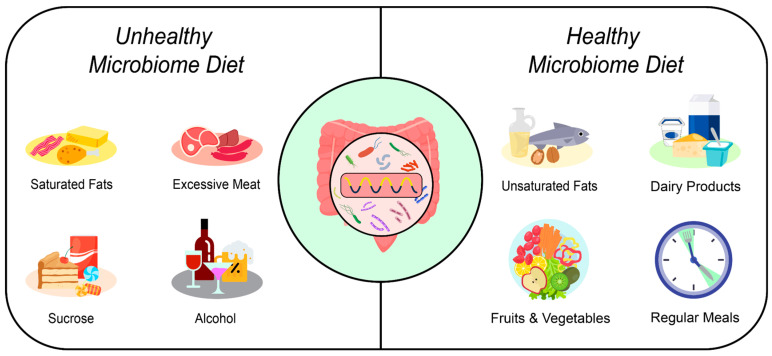
The influence of diet on the gut microbiota.

Dietary habits significantly shape the composition of the gut microbiota. Key factors include the quality of the diet, the timing of food intake, the regularity of meals, and the intervals between meals, all of which influence the microorganisms residing in the intestines [33,35,141] (Figure 3). These factors have been extensively studied and are well-documented in the scientific literature.

#### 3.5.1. Timing of Meals and Intervals between Eating Episodes

The food entering the gastrointestinal tract serves as a primary synchronizer of the peripheral clocks located within it. Consequently, consumption of food in the late evening may have adverse effects on health, increasing the risk of serious complications such as alterations in hormone secretion, disruption of circadian rhythms, and changes in the composition of the gut microbiota [32,142]. The human body usually operates in two main phases: an active phase that begins around 10 a.m. and a rest phase that commences at 10 p.m. [33]. The regularity of these phases is influenced by fluctuations in hormone production, which predominantly occurs during the active phase [143]. A delay in the final meal of the day by even an hour has been shown to result in increased levels of *C*-reactive protein, insulin, glucose, and glycated hemoglobin, as well as a decrease in HDL cholesterol levels [144]. Moreover, it has been demonstrated that consuming meals earlier in the day is associated with greater weight loss [145]. Furthermore, there is evidence to suggest that time-restricted eating is associated with alterations of the gut microbiota. Restricting food access to specific times has been observed to result in an increase in the presence of beneficial bacteria such as *Oscillibacter* and *Ruminococcaceae*, and a reduction in the population of *Lactobacillus* and *Lactococcus* [142]. Furthermore, individuals who consume meals late in the day, particularly after 2:00 p.m., tend to have higher levels of Lachnospira, which is associated with the consumption of more calories during these later hours [146,147].

In addition to meal timing, the frequency and regularity of eating are also critical considerations. Frequency refers to the number of meals consumed during the day, while regularity is an indicator of their consistency [148,149]. The circadian rhythm of the host may be affected by the consumption of meals at irregular times or by the omission of meals altogether [33]. For instance, individuals with an evening chronotype, who typically experience shifted sleep schedules, are more likely to skip breakfast. This tendency can be attributed to their habit of accumulating sleep debt, which often leads them to extend their morning sleep, frequently at the expense of breakfast [33].

#### 3.5.2. Dietary Fiber

The type, quality, and origin of the food consumed shape the gut microbiome and influence its composition and function. These factors, in turn, affect host–microbiome interactions. In a diet typical of a Western lifestyle, there is a marked deficiency in the intake of complex carbohydrates, which are important sources of dietary fiber. This deficiency can irreversibly reduce microbial diversity, leading to the disappearance of certain microbial species in the digestive system [53,150]. Characteristic aspects of this diet, such as an increased intakes of sugar and saturated fat and a reduced intakes of dietary fiber, may contribute to the higher prevalence of chronic diseases such as type II diabetes, cancer, obesity, and inflammatory bowel disease [53,151].

As defined by the 2009 Codex Alimentarius Commission in 2009 [152], dietary fiber comprises edible carbohydrate polymers consisting of ten or more monomeric units that are resistant to endogenous digestive enzymes and, thus, are not hydrolyzed or absorbed in the small intestine [48,153,154]. Dietary fiber is characterized by great diversity, so its description is based on various classifications, including origin, chemical composition, and physicochemical properties, with an additional subdivision based on the degree of polymerization [153]. Dietary fiber can also be classified by its source of origin, with common sources including cereals, vegetables, fruits, nuts, seeds, legumes, and pulses. Fibers from different plant groups have varying chemical compositions that may interact favorably with the diverse composition of the gut microbiota. It is, therefore, important to maintain a varied diet that includes a range of plant foods, rather than merely focusing on the amount of fiber alone [152,153,154].

Dietary fiber can also be classified according to their solubility. Insoluble fibers are bound together by tight hydrogen bonds, forming a crystalline and hydrophobic structure that is resistant to hydrolysis by exogenous glucosidases. Common types of insoluble dietary fiber include cellulose, hemicellulose, and lignin. These fibers contribute to the bulkiness of stool material, but are less readily utilized by gut microorganisms than soluble fiber [154,155]. As a result, soluble dietary fiber is readily metabolized more readily by gut microorganisms, and, thus, significantly influences the abundance and diversity of the human gut microbiota [153,155]. Once soluble fiber reaches the colon, it undergoes transformation through various degradative mechanisms into oligo- and monosaccharides, which are then absorbed by specific transport systems to derive energy [156]. The degradation of dietary fiber by the gut microbiota results in the production of organic acids, gases, and a significant amount of SCFAs [154,157].

The production of short-chain fatty acids has many benefits for the body, including reduced risks of gastrointestinal diseases, cancer, and inflammatory conditions, as well as alleviating functional constipation and improving sleep quality [150,158]. Moreover, most types of soluble fibers encourage the proliferation of microorganisms in the intestines, which is why they are referred to as prebiotics [49,159]. Thus, consuming a sufficient amount of fiber-rich food can positively influence the diversity and abundance of gut microorganisms [154]. However, chronic or periodic fiber deficiency, may result in the secretion of mucosal glycoproteins as a nutrient source, potentially leading to erosion of the colonic mucosal barrier [160]. This, in turn, might result in intestinal barrier dysfunction, which can escalate to fatal colitis [160]. Ensuring a varied diet that incorporates a range of plant-based products in every meal is crucial for maintaining overall health [53,153,161,162]. It is recommended to consume foods rich in omega-3 fatty acids and unsaturated fatty acids, while limiting saturated fatty acids.

#### 3.5.3. Polyphenols

Polyphenols are organic chemical compounds that occur naturally in plants. They are found in a wide variety of foods, including fruits, vegetables, grains, tea, coffee, wine, nuts, seeds, spices, legumes, and oils [163,164]. Although characterized by their phenolic structural features, polyphenols show considerable diversity in structure, which is the basis for their classification into several subgroups. They are categorized on the basis of the number of phenolic rings and the structural elements linking these rings. The major structural groups of polyphenols are flavonoids, phenolic acids, lignans, and stilbenes [164,165]. The extensive literature highlights the importance of polyphenols in numerous biological functions, including their anti-inflammatory, immunomodulatory, anticancer, antidiabetic, cardioprotective, neuroprotective, and gastroprotective properties [166,167,168]. Polyphenols and their active metabolites also stimulate the production of SCFAs and branched-chain amino acids, which may be beneficial in the treatment and prevention of various diseases, and particularly gastrointestinal disorders [51].

Concepts such as bioavailability and bioactivity are used to assess the efficacy of polyphenols [169,170]. Only a small proportion of polyphenols can be directly absorbed in the small intestine. More complex polyphenols are transformed in the large intestine by the gut microbiota, which converts them into low-molecular-weight metabolites [171]. These unabsorbed, complex polyphenols that reach the large intestine may influence the composition of the gut microbiota, thereby exerting a beneficial effect on host health [171]. The secondary metabolites produced by the microbial metabolism of polyphenols can act as prebiotic molecules, capable of modifying the growth of specific microbial strains [171].

Polyphenols can interact with the gut microbiota, and act as prebiotics to enhance the proliferation of various microbial strains, thereby positively influencing the composition of the gut microbiota. As polyphenols are not digested by host digestive enzymes, they can serve as substrates for gut microorganisms [172,173]. This property allows polyphenols to significantly increase the proliferation of health-promoting bacterial strains, such as *Bifidobacterium* and *Lactiplantibacillus*, while inhibiting the growth of harmful species such as *Clostridium* and *Escherichia coli* [174]. 

The mechanisms by which polyphenols interact with the microbiota are still unclear. However, they may have both direct and indirect effects. For instance, polyphenols can directly stimulate or inhibit the growth of certain bacteria. In the case of stimulation, resistance is closely linked to the bacteria’s ability to metabolize these compounds, whereas in the case of inhibition, the antimicrobial properties of these compounds play a crucial role [175,176]. In addition, polyphenols may have various positive effects, such as anti-inflammatory, antioxidant, immunomodulatory, anticancer, and cardiovascular protective effects [170,177]. Therefore, the supply of these compounds from plant foods is essential for maintaining the abiotic state within the gut ecosystem and the overall health of the organism.

#### 3.5.4. The Quality and Quantity of Fats

The quality and quantity of fats affect the gut microbiota. These include saturated fatty acids, monounsaturated fatty acids, and polyunsaturated fatty acids, which are classified according to the presence of double bonds between carbon atoms [178]. Animal products are the main source of saturated fatty acids, while unsaturated fatty acids are mainly derived from plant products.

Excessive dietary intake of saturated fatty acids may be associated with a decrease in *Bacteroidetes* and an increase in *Firmicutes* and *Proteobacteria*, as observed in several animal studies [179,180]. This shift may lead to disturbances in the intestinal barrier [181]. Conversely, the consumption of omega-3 polyunsaturated fatty acids may affect the gut microbiota by modifying its composition and abundance, regulating the concentration of SCFAs and altering the concentration of pro-inflammatory mediators [182]. High consumption of fish oil, which is a major source of omega-3 fatty acids, may result in reduced growth of *Enterobacteria* and increased growth of *Bifidobacteria*, thereby favoring the gut microbiota [183]. In addition, omega-3 polyunsaturated fatty acids may contribute to an increased thickness of the intestinal mucosal layer, improving the intestinal microenvironment, and enhancing the function of the mucosal barrier [184].

Moreover, omega-3 fatty acids can increase the production of SCFAs, which have potent anti-inflammatory properties and are essential for maintaining gut health [182,185]. Consumption of monounsaturated fatty acids, such as extra virgin olive oil, has also been shown to have beneficial effects, in particular increasing the diversity of the gut microbiota. Interestingly, this olive oil is rich in polyphenols, which have a positive effect on the gut microbiota [186,187]. Consuming products rich in omega-3 polyunsaturated fatty acids, unsaturated fatty acids, and limiting saturated fatty acids may have a beneficial effect on the gut microbiota.

#### 3.5.5. Sucrose and Fructose

Excessive sugar intake significantly impacts the gut microbiome, leading to various health-related changes. A diet high in sugars, such as sucrose and fructose, can impair the permeability of the intestinal walls and alter the composition of the gut microbiota [188,189]. The Western diet, rich in sugar and fat, can affect the gut microbiota within one day [190]. In individuals following this diet, a decrease in Bacteroides and an increase in Firmicutes can be observed [191]. In contrast to fiber-rich diets, the Western diet leads to a decrease in the production of SCFAs and impairs the intestinal barrier [192,193]. High sugar intake can also result in an increase in Proteobacteria [193]. Additionally, it can lead to a decrease in Prevotella [193], which is also observed in patients with chronic insomnia [58].

#### 3.5.6. Excessive Meat Consumption

Meat overconsumption, especially red and highly processed meat, can result in an increase in the numbers of Firmicutes and Proteobacteria [194]. Additionally, a diet high in animal fats can contribute to decreased production of SCFAs and lipopolysaccharides, leading to systemic low-grade inflammation [16,195]. Optimal production of SCFAs in the body is a factor that can affect sleep quality [158]. These acids can influence the production of TPH1, thereby contributing to increased serotonin synthesis [45,66]. High consumption of processed foods, including meat, can also increase intestinal permeability [196].

#### 3.5.7. Alcohol

The consumption of alcoholic beverages has a negative impact on the functioning of the gut microbiome [197,198]. Alcohol consumption by pregnant women can affect the newborn’s gut microbiota [198,199]. Among newborns whose mothers consumed alcohol during pregnancy, an increase in the number of Megamonas has been observed, which can influence later gut colonization [200]. Both chronic and acute alcohol consumption can lead to specific changes in the gut microbiome [201]. Alcohol consumption can increase the numbers of Actinobacteria and Proteobacteria while decreasing the numbers of Firmicutes [202]. Chronic alcohol use can lead to a decrease in Bacteroidetes and an increase in Proteobacteria. Additionally, it may lead to negative changes in the intestinal barrier, including increased intestinal permeability [198,203].

## 4. Strengths and Limitations

A thorough search of the PubMed database was conducted, making every effort to locate all systematic reviews and meta-analyses related to the discussed topics. The article analyzes the relationship between dietary diversity and its effects on the gut microbiome, the production of certain metabolites, and sleep quality. Additionally, it examines how sleep disturbances and gut microbiota can contribute to the occurrence of complications. The aim was to provide an up-to-date and in-depth analysis of the literature, gathering data and methodologies that have shaped previous findings and the current understanding of the research.

One limitation of this work is its reliance on the available literature, which often comprises studies conducted on relatively small participant groups. To draw more reliable and comprehensive conclusions, further research involving larger participant numbers is needed to better understand the impact of gut microbiota and diet on sleep.

Additionally, while this article reviews current knowledge on the influence of gut microbiota on sleep quality, more detailed experimental studies are necessary to explore these issues across different populations and contexts.

Finally, the varied methodologies used in studies on the gut microbiome and diet present challenges in comparing results and forming clear conclusions. These methodological differences include variations in measuring food intake, techniques used to analyze the microbiome, and the diverse population groups studied.

## 5. Summary

Research indicates that the gut and brain are connected through the gut–microbiome–brain axis, with the gut microbiota influencing the brain via immunoregulatory, neuroendocrine, and vagus nerve pathways [21,22]. The microorganisms colonizing the human gut produce various metabolites, including neurotransmitters capable of affecting the nervous system. The production of metabolites by microorganisms occurs cyclically, playing a crucial role in regulating the host’s circadian rhythms and metabolism. This additional evidence suggests a significant influence of the microbiota on metabolic homeostasis and the organism’s rhythmicity [26,28].

Dietary fiber is fermented in the colon by the gut microbiota, resulting in the production of SCFAs. Studies indicate that certain bacterial strains including *Lachnospiraceae* UCG004 and *Odoribacter*, involved in SCFA production, contribute to longer sleep duration [51]. SCFAs potentially impact sleep by modulating the synthesis of GABA and 5-HT. GABA, the primary inhibitory neurotransmitter in the nervous system, plays a significant role in promoting sleep by inhibiting arousal pathways [42,63]. 

Serotonin production in the gut depends on the gut microbiota, diet, host signaling hormones, and peptides [45]. The gut microbiota may play a key role in supporting optimal melatonin production, significantly impacting sleep regulation and other circadian-related functions [75,76,79].

Nutrition has a significant impact on the gut microbiota. Factors influencing the microbiota composition include diet quality, meal timing, and its regularity [33,35]. Studies have shown that earlier meal times positively affect metabolic homeostasis and circadian rhythms [35,142]. Furthermore, a diet rich in healthy fats, such as monounsaturated fatty acids found in olive oil and omega-3 polyunsaturated fatty acids present in fish oil, can positively influence the gut microbiota [182]. In addition, regular consumption of dietary fiber found in various plant products and polyphenols naturally occurring in plants can support the diversity and functions of the gut microbiota, contributing to overall health [53,153,171,173]. Therefore, a balanced diet consisting of wholesome meals eaten regularly, containing healthy fats, fiber, and polyphenols, is essential for maintaining a healthy gut microbiota and overall health and consequently sleep quality.

## 6. Conclusions

Consuming a varied diet comprising foods abundant in dietary fiber, polyphenols, and unsaturated fatty acids can exert a favorable impact on the gut microbiome, potentially influencing sleep patterns. Metabolites synthesized by the microbiota, such as short-chain fatty acids (SCFAs), gamma-aminobutyric acid (GABA), serotonin (5-HT), melatonin, and tryptophan, play crucial roles in modulating physiological functions, including sleep regulation. Imbalances in microbiota composition may be associated with the onset of sleep disorders as well as other chronic conditions, including obesity, cardiovascular diseases, and mental disorders.
